# Prediction of pathological response following neoadjuvant chemotherapy in patients with muscle-invasive bladder cancer: the PRE-PREVENCYS trial

**DOI:** 10.1186/s12885-021-08840-2

**Published:** 2021-10-29

**Authors:** F. J. Hinsenveld, B. J. Noordman, J. L. Boormans, J. Voortman, G. J. L. H. van Leenders, S. L. van der Pas, S. C. van Beek, D. E. Oprea-Lager, A. N. Vis

**Affiliations:** 1 Department of Urology, Amsterdam University Medical Centers, VU University, Postbus 7057, 1007, MB Amsterdam, internal post address 4F-28 The Netherlands; 2 Department of Surgery, Erasmus MC, University Medical Center Rotterdam, Rotterdam, The Netherlands; 3 Department of Urology, Erasmus MC, University Medical Center Rotterdam, Rotterdam, The Netherlands; 4 Department of Medical Oncology, Amsterdam University Medical Centers, VU University, Amsterdam, The Netherlands; 5 Department of pathology, Erasmus MC, University Medical Center Rotterdam, Rotterdam, The Netherlands; 6 Department of Epidemiology and Biostatistics, Amsterdam University Medical Centers, VU University, Amsterdam, The Netherlands; 7 Department of Radiology & Nuclear Medicine, Cancer Center Amsterdam, Amsterdam University Medical Centers, VU University, Amsterdam, The Netherlands

**Keywords:** Bladder cancer, Cancer biomarkers, Cystectomy, Neoadjuvant chemotherapy, Residual tumour, Bladder-sparing

## Abstract

**Background:**

The recommended treatment for patients with non-metastatic muscle-invasive bladder cancer (MIBC) is neoadjuvant chemotherapy (NAC) and radical cystectomy (RC). Following NAC, 20–40% of patients experience a complete pathological response (pCR) in the RC specimen and these patients have excellent long-term overall survival. Subject to debate is, however, whether patients with a pCR to NAC benefit from RC, which is a major surgical procedure with substantial morbidity, and if these patients might be candidates for close surveillance instead. However, currently it is not possible to accurately identify patients with a pCR to NAC in whom RC might be withheld. The objective of this study is to assess whether pathological response in the RC specimen after NAC can be predicted based on clinical, radiological, and histological variables and on a wide set of molecular biomarkers assessed in tissue, blood and urine.

**Methods:**

This is a multicentre, prospective cohort study, including patients with cT2a-T4a N0-N1 M0 urothelial cell MIBC who are scheduled to undergo cisplatin-based NAC followed by RC. Prior to start of therapy, a 2-Deoxy-2-[18F] fluorodeoxyglucose (^18^F-FDG) positron emission tomography/computed tomography (PET/CT) is performed. Response to NAC is evaluated by CT-scan. Blood and urine, including cytology, are prospectively collected for biomarker analyses before and after NAC. Immediately before RC, participants undergo cystoscopy with bimanual examination and a re-staging transurethral resection (TUR) of all visible cancerous lesions or with biopsies from scar tissue. Subsequently, RC is performed in all patients. Tissue from the diagnostic TUR, the re-staging TUR, and the RC specimen is examined for the presence of urothelial cancer carcinoma and DNA and RNA is isolated for molecular analysis. The primary endpoint is the pathological stage (ypTN) in the RC and ePLND specimen and its association with clinical response.

**Discussion:**

If the PRE-PREVENCYS trial shows that the absence of residual disease after NAC in patients with MIBC is accurately predicted, a randomized controlled trial is scheduled comparing the overall survival of NAC plus RC versus NAC followed by close surveillance for patients with a clinically complete response (PREVENCYS trial).

**Trial registration:**

Netherlands Trial Register: NL8678; Registered 20 May 2020 https://www.trialregister.nl/trial/8678

## Introduction

### Background

Muscle-invasive bladder cancer (MIBC) is an aggressive disease with a 5-year mortality rate of 40–50% [1]. Radical cystectomy (RC) with extended pelvic lymph node dissection (ePLND) and urinary diversion is considered the cornerstone for curative treatment in patients with MIBC [2]. However, RC is a major surgical procedure that is associated with a 90-day major morbidity rate up to 22% and a 90-day mortality rate of 2 to 8% [2–5]. In 2016, 962 patients underwent RC in the Netherlands in 41 hospitals [1]. Randomized clinical trials (RCTs) demonstrated a 5-year overall survival benefit of 8% for patients with non-metastatic MIBC undergoing cisplatin-based neo-adjuvant chemotherapy (NAC) before radical surgery compared to those who did not undergo NAC [6–8]. Consequently, international guidelines recommend NAC prior to RC in all patients with cT2–4aN0–1 M0 MIBC who are fit for cisplatin chemotherapy [2]. Interestingly, it is observed that following NAC, approximately 20 to 40% of patients have no residual urothelial cancer cells on histological examination of the RC specimen and on ePLND [9–11]. These patients with a pathologically complete response (pCR) to NAC have excellent outcomes with reported 5-yr overall survival rates of 80–85% [9]. It is even hypothesized that patients with a pCR after NAC might not benefit from concurrent RC as there is no residual tumour present in the bladder. The 10–15% of patients who develop distant metastases early after NAC might also not benefit from removal of the bladder as these patients had chemotherapy-refractory subclinical metastases at the time of RC. Especially those with pCR after NAC may be suitables candidates for close surveillance by frequent diagnostic investigation of the bladder and pelvic lymph nodes. By close active surveillance, tumour recurrences may be detected in an early and treatable stage of disease. In case of locoregional recurrence of MIBC, without signs of distant metastases, the patients can undergo (postponed) RC. Non-muscle invasive recurrences may be treated by intravesical therapies or repeated transurethral resections. Such close surveillance strategies have been shown to be feasible in oesophageal and rectal carcinoma [12, 13].

### Objective

For now, it is not yet possible to adequately predict the histopathological characteristics, such as pCR, in the RC specimen based on routine clinical, biochemical, radiological and histopathological variables determined preoperatively. Today, a lot of scientific research is performed on the predictive value of molecular biomarkers that may be determined in the urine and blood of patients with MIBC or in the resected bladder cancer tissue, for example DNA damage response (DDR) genes ATM, RB1, FANCC, ERBB2, and ERCC2 [14–17]. These molecular biomarkers have been associated with tumour aggressiveness features and with patient prognosis but have not been adapted into predictive models along conventional prognostic factors.

In the present prospective, single arm, multicentre study, we aim to predict the histopathological characteristics in the RC specimen using a wide set of clinical, radiological, histopathological and molecular biomarkers. A specific aim is to identify patients in whom a RC could be withheld or postponed because of a high preoperative estimate of pCR**.** These patients may be potential candidates for a bladder cancer sparing approach and concurrent close active surveillance. Bladder sparing treatment has a substantial positive impact on the quality-of-life of these patients who were previously scheduled for radical surgery. Theoretically, a nonsurgical treatment strategy in those patients with a complete response after NAC will save up to 4% mortality, 22% of major morbidity and, potentially, reduces health care costs. The acronym of this trial is the PRE-PREVENCYS trial (‘prevent cystectomy’). The results obtained with this preparatory study will help us estimate the number of patients needed for a subsequent RCT. This future so-called PREVENCYS trial will randomize patients with a clinically complete response, i.e.*,* those without signs of residual disease after NAC based on a predictive algorithm including molecular biomarkers obtained with the PRE-PREVENCYS trial, into two study arms, i.e., [1] NAC plus RC, and [2] NAC followed by a close active surveillance protocol.

## Methods

### Study design

The PRE-PREVENCYS trial is a prospective multicentre cohort study including 180 MIBC patients. Currently, eight high-volume medical centres, both academic and general hospitals, are participating: Amsterdam University Medical Centres, Erasmus Medical Centre Rotterdam, Netherlands Cancer Institute Antoni van Leeuwenhoek Amsterdam, Radboud University Medical Centre Nijmegen, Canisius-Wilhelmina Hospital Nijmegen, Rijnstate Hospital Arnhem, University Medical Centre Utrecht and St. Antonius Hospital Nieuwegein. The study was created using the Transparent reporting of a multivariable prediction model for individual prognosis or diagnosis (TRIPOD) statement and the REporting recommendations for tumour MARKer prognostic studies (REMARK) guideline [18, 19].

### Study population

The following inclusion criteria were used:

- Adult patients,
- Histologically proven locally confined or locally advanced MIBC, i.e., cT2-T4a 0-N1M0,
- Predominantly urothelial cell bladder carcinoma,
- Scheduled for cisplatin-based NAC prior to RC with extended pelvic lymph node dissection (ePLND),
- Prior to NAC, no evidence of regional or distant metastases on staging 2-Deoxy-2-[18F] fluorodeoxyglucose (^18^F-FDG) position emission tomography/computed tomography (PET/CT), albeit a single node in the surgical template of the ePLND (cN0–1 M0) is allowed

The decision to perform NAC followed by RC, either open or robot-assisted, with ePLND and urinary diversion is determined by a local multidisciplinary tumour board. The decision to give dose-dense Methotrexate, Vinblastine, Adriamycin and Cisplatin (ddMVAC) every two weeks or Gemcitabine and Cisplatin (Gem/Cis) every three weeks as cisplatin-based NAC is determined by the medical oncologist according to local hospital protocols. Patients with a Creatinine Clearance (CrCl) between 40 and 60 ml/min are eligible for split-dose cisplatin and gemcitabine [20].

The following exclusion criteria were used:

- Carcinoma in situ (CIS) in the urethra prostatica at diagnosis
- Patients with concomitant tumours of the upper urinary tract, tumours of the urachus or an additional malignancy that is progressing or has required active treatment within the past three years are excluded. Exceptions to these include patients with basal cell carcinoma of the skin, squamous cell carcinoma of the skin that has undergone potentially curative therapy, or carcinoma in situ (e.g., breast carcinoma in situ, cervical carcinoma in situ), who have undergone potentially curative therapy. Participants with low-risk early-stage prostate cancer defined as follows will not be excluded: Stage T1c or T2a with an International Society of Urological Pathology (ISUP) grade 1 and prostate-specific antigen < 10 ng/mL either treated with definitive intent or on active surveillance that has been stable for the past year prior to study allocation.
- Clinical response evaluation (CRE) by CT scanning during NAC shows progression of local disease or pulmonary, osseous, hepatic, or non-regional lymph-node metastases.

### Study algorithm (Table 1, Fig. 1)

#### Overview

At baseline, a staging ^18^F-FDG-PET/ diagnostic CT for attenuation and anatomical correction, from vertex to mid-thigh, is performed. Patients with absence of metastatic disease (cN0–1 M0) on FDG-PET/CT, are scheduled for cisplatin-based NAC followed by RC with ePLND. Prior to start of NAC, and right before or after the ^8^F-FDG-PET/CT, patients undergo the first liquid biopsy collection of blood and urine. During NAC, a standard clinical response evaluation (CRE) with a diagnostic CT scan of the thorax and abdomen is performed. All patients with stable disease or partial or complete radiological response without new suspicious lesions at CRE during NAC, continue to participate in the PRE-PREVENCYS trial. Patients who complete less than three cycles of NAC will be excluded for the per-protocol analyses but will continue in the PRE-PREVENCYS trial for the intention-to-treat analyses. Second liquid biopsy collection of blood and urine will be scheduled at hospital admission for surgery. Patients will undergo bimanual examination, cystoscopy and a re-staging transurethral resection (TUR) of all visible lesions and of scar tissue under anaesthesia during RC. As explained above, the aim of the liquid biopsies before and after NAC and the re-staging TUR prior to RC is to investigate whether response to NAC can be predicted using clinical, radiological, and histological variables as well as a wide set of predictive molecular biomarkers in tissue, blood and urine.

**Table 1:** Overview of study visits for participants of the PRE-PREVENCYS trial

	Pre-treatment			NAC^e^ week 3–15		Surgery	Follow-up
Visit	Visit 0	Visit 1	Visit 2	Visit 4	Visit 5	Visit 6	Visit 7
Week	-2	0	2	7–8 or 11–12	15–16	20–22	32–35
Informed consent	X*						
Inclusion		X*					
ECOG performance status		X					
Liquid biopsy^a^		X*				X*	
Urine cytology		X				X*	
Blood hematology^b^ and biochemistry^c^		X				X	
Staging ^18^F-FDG-PET/CT^d^			X				
CRE1^f^				X			
(optional) CRE2^f^					X		
BME, cystoscopy and TUR^g^						X*	
RC with ePLND						X	
CRE3^f^							X

^a^ Collection of blood and urine for biomarker analyses

^b^ Hematology: complete blood count, white blood differential, CRP

^c^ Biochemistry: serum albumin, electrolytes, serum creatinine, bilirubin, alkaline phosphatase, AST, ALT, LDH

^c^ FDG PET/CT of thorax and abdomen for staging purposes

^e^ At least three cycles of cisplatin-based NAC, i.e. 3w Gemcitabin/Cisplatin or 2w dose-dense Methotrexate, Vinblastine, Adriamycin and Cisplatin

^f^ CRE: CT scanning of thorax and abdomen. CRE1 after three cycles of NAC; optional CRE1 after two cycles of NAC in combination with an additional CRE2 after completion of NAC; and CRE3 at three months post-surgery

^g^ Prior but in the same session of RC at the operation room. TUR of lesions suspected for tumour, or scar tissue

* Study-related actions or interventions

BME: bimanual examination; CRE: clinical response evaluation; CT: computed tomography; ECOG: Eastern Cooperative Oncology Group; ePLND: extended pelvic lymph node dissection; ^18^F-FDG: 2-Deoxy-2-[18F] fluorodeoxyglucose; NAC: neoadjuvant chemotherapy; PET: positron-emission tomography; RC: radical cystectomy; TUR: transurethral resection

**Fig. 1:**
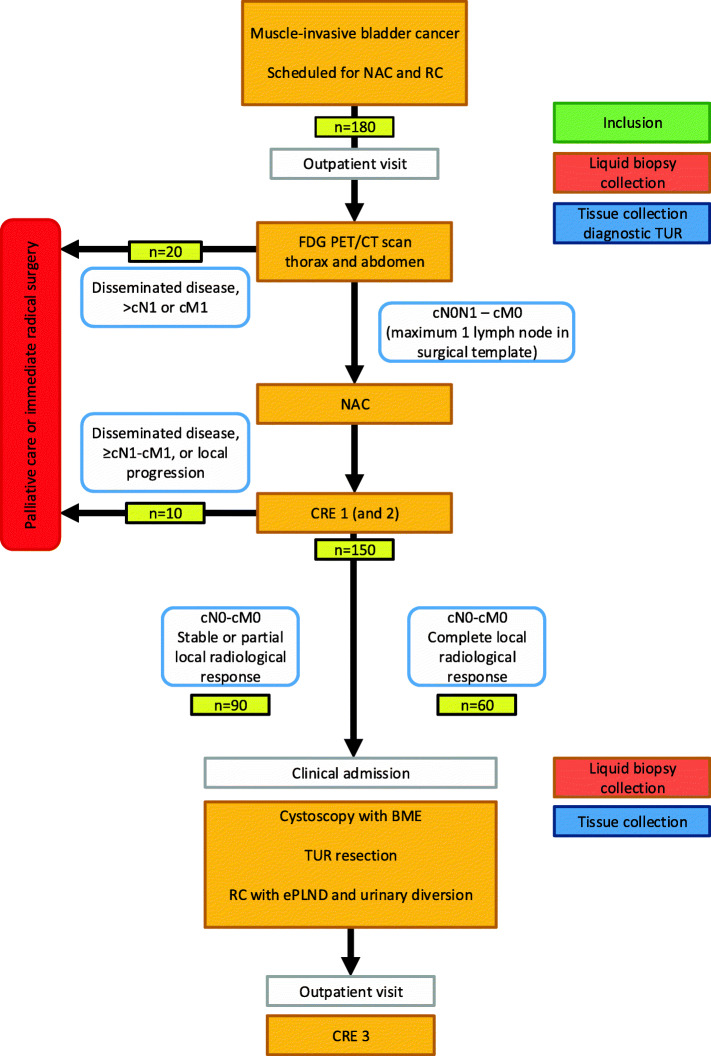
Study algorithm of the PRE-PREVENCYS trial with expected distribution of patients. BME: bimanual examination; CRE: clinical response evaluation; CT: computed tomography; ddMVAC: dose-dense Methotrexate, Vinblastine, Adriamycin and Cisplatin; ePLND: extended pelvic lymph node dissection; FDG: 18F-fluoro-2-deoxy-D-glucose; Gem/Cis: Gemcitabine/Cisplatin; NAC: neoadjuvant chemotherapy; PET: positron-emission tomography; RC: radical cystectomy; TUR: transurethral resection

#### Liquid biopsies

Liquid biopsy collection of blood and urine will coincide with blood collections scheduled for routine care. Time points for liquid biopsy collection are: 1) at baseline, before start of NAC, and 2) upon completion of NAC, i.e., prior to surgery. Blood samples and urine specimens will be collected and processed to allow assessment of liquid biopsy-based biomarkers including circulating tumour DNA (ctDNA), extracellular vesicle (EV)-based biomarkers (e.g., microRNAs) and platelet-based biomarkers (e.g., mRNA) (Protocols available upon request). Simultaneously with both liquid biopsy collections, blood haematology and biochemistry, and urine cytology will be collected.

#### Clinical response evaluation during NAC

Patients will be re-evaluated at least once, optionally twice, by standard CRE(s) during NAC and before undergoing surgical resection. The aim of these CRE(s) will be to identify those patients in whom progression of local disease, absence of a local response and/or disseminated disease is present. All visits within CREs will be part of standard clinical care and will follow local hospital protocols. In case only one CRE is performed (CRE1), a CT scan with intravenous contrast of thorax and abdomen will be performed approximately 1–2 weeks after the third cycle of NAC. In case the patient will be scheduled for two CREs (CRE1 and CRE2), a CT scan is performed approximately 1–2 weeks after the second cycle of NAC and approximately 1–2 weeks after the completion of all NAC courses. Eligibility of the patients for continuation in the PRE-PREVENCYS trial will be assessed based on the findings of the evaluation CT scan after three or four cycles of NAC, using the RECIST 1.1 criteria [21]. In absence of disseminated disease or local progression at CRE, patients will be scheduled for RC, ePLND and urinary diversion approximately four to six weeks after the last course of NAC.

#### Surgery

On the day of RC, a cystoscopy of the bladder will be performed with the patients under general anaesthesia. This is different than regular care. During this procedure, bimanual examination and a TUR of any visible lesions suspected for residual tumour within the bladder and of the scar tissue from the previous location of the bladder tumour will be performed. This re-staging TUR should include at least one biopsy including detrusor muscle but does not have to be a radical resection of the lesion. In case of absence of tumour lesions and scar tissue, random biopsies will be taken of different locations of the bladder including one of the location of the previous bladder tumour. The diameter of the biggest visible lesions will be reported, as well as the estimated tumour-stage by the urologist and the duration of the re-staging TUR. The aim of the TUR biopsies is to determine the accuracy of a re-staging TUR in the detection of residual disease after NAC. Subsequently, open or robot-assisted RC will be performed with ePLND and urinary diversion as per local protocol.

#### Follow-up

Approximately three months after RC, the first follow-up visit will be planned in concordance with local follow-up protocols. A diagnostic CT scan of thorax and abdomen will be performed to stage for recurrence of disease (CRE3). This is standard of care.

#### Radiology

All ^18^F-FDG PET/CT scans and diagnostic CT thorax/abdomen scans during CRE(s) will be revised centrally by a specialized nuclear physicist and radiologist, respectively. Clinicians are blinded for the pathology outcome in the RC specimens or the TUR tissue material. The CT scans during CRE(s) will be revised according to the RECIST 1.1 criteria [21].

#### Pathology

The following formalin-fixed, paraffin-embedded (FFPE) tissue specimens will be examined in the current study: 1) the diagnostic TUR, 2) the re-staging TUR of residual bladder tumour or scar tissue after NAC, collected in the scope of the trial protocol, and 3) the diagnostic RC and ePLND specimens. All tissue specimens will be revised centrally by an expert uropathologist. Tumour type grade (WHO 1973 and 2016), variant histology, aberrant differentiation, concomitant CIS, ypT-stage (Union for International Cancer Control, 8th edition), surgical margin status (R0 or R1) and lymph node status (pN0 or pN1) will be monitored [22]. Response to chemotherapy in both the RC and PLND specimens will be assessed according to the Tumour Regression Grade (TRG) ranging from 1 (pathological complete response) to 3 (weak/ no response) [23]. The pathologist will be blinded to the outcome of radiological imaging such as those of CRE1 and CRE2. For revision and histopathological evaluation of the study re-staging TUR biopsies, the pathologist will be blinded to the histopathological outcome of the RC resection specimen as well. FFPE tissue samples of all three time points will be collected for genomic, gene expression, and immunohistochemical studies for biomarker analyses.

### Biomarker analyses

DNA and RNA will be isolated form cancerous regions of the TUR and RC specimens containing at least more than 70% tumour cells. In addition, DNA will be isolated from normal non-malignant adjacent tissue for germline DNA. After quality control, whole exome sequencing (WES) and RNA sequencing will be done and analysed. By WES of diagnostic TUR samples [24], we will assess the proportion of mutated DDR genes and loss of mismatch repair (MMR) genes, and tumour mutational burden (TMB) in our cohort of patients with a radiological response at CRE during NAC and we will correlate the molecular alterations with the pathological response to NAC at RC. By RNA sequencing analysis we will validate molecular subtypes in our cohort of patients with a radiological response at CRE during NAC and correlate them with the pathological response to NAC at RC. In addition, unsupervised hierarchical clustering will be done to identify differentially expressed genes between responders and non-responders. Furthermore, immunohistochemical staining studies with antibodies will be performed.

Based on results from tissue biomarker studies, candidate prognostic and predictive DNA and RNA biomarkers will be defined and subsequently assessed at both liquid biopsy time points by quantitative Polymerase Chain Reaction (qPCR) in order to investigate whether a liquid biopsy-based test of blood or urine correlates with or can complement or replace a tissue-based DNA/RNA biomarker test. Transcriptome analysis of tumour-educated blood platelets (TEP) collected pre-treatment will be performed in order to establish bladder cancer specific TEP-RNA signatures as well as prognostic and predictive signatures [25, 26]. Extracellular vesicle next generation microRNA (EV-microRNAs) sequencing will be performed. A discovery cohort consisting of 10 patients with a pCR following NAC versus 10 patients with an incomplete or no response will first be analysed. For these 20 patients EV-RNA sequencing will be performed using both urine and plasma specimens collected at baseline and post chemotherapy. Candidate biomarkers will be established for treatment outcome prediction. Validation by quantitative reverse transcription PCR (RT-qPCR) of the most promising candidate miRNA panels will be performed with Taqman assays. Based on prior studies we expect to minimally detect 10 potential candidate markers that can be used for further evaluation and technical validation. Ultimately, we will strive for an assay that measures three miRNAs. Such small panels can reach high levels of specificity and sensitivity, although validation in independent collected cohorts will be of importance.

Additionally, isolated EV fractions will be used as an alternative bio-specimen source for qPCR of other potential DNA and RNA biomarkers based on tissue-based biomarker studies. These results will then be compared to the unfractionated plasma and urine DNA/ RNA qPCR results in order to determine the optimal bio-specimen for liquid biopsy-based biomarker assessment.

### Study parameters/endpoints

The main endpoint of this study is the final pathological stage after NAC (i.e., ypT and ypN stage), determined in the RC and ePLND specimen. Secondary endpoints are:

- The number of patients with pCR after NAC. pCR is defined as the absence of tumour cells in the RC specimen and in the resected pelvic lymph nodes (ypT0N0) or the presence of a non-invasive papillary tumour in the bladder without tumour cells in the resected pelvic lymph nodes (ypTaN0).
- The number of participants in whom RC could have been withheld, i.e., the number of patient who could have undergone a bladder-sparing approach, if imaging, urine cytology, histological examination on re-staging TUR and molecular biomarker analyses was not followed by RC.
- Perioperative complications of the re-staging TUR, such as perforation of the bladder wall, major bleeding in the bladder where haemostasis is not achieved with coagulation alone or any other surgical complications directly related to the re-staging TUR.
- The number of participants who had ≥cN2Mx or cNxM1 disease on the staging FDG-PET/CT and were therefore counselled for a different treatment strategy than NAC plus RC.

### Interim analysis and (serious) adverse events

Adverse events are defined as any undesirable experience occurring to a subject during or within the first 24 h after re-staging TUR performed at the time of RC. These adverse events need to be directly associated with the TUR during RC, such as major bleedings in the bladder, bladder perforation during TUR and other surgical complications directly related to TUR. Any minor complications, such as small bleeders occurring during re-staging TUR that can be easily coagulated, are not assessed as adverse event. As the urinary bladder is removed in the same procedure, none of these minor complications is expected to have a significant influence on patient outcome or burden. Any adverse events will be reported to the principal investigator (AV) within 1 week of occurrence. An interim analysis is not foreseen.

### Statistical analyses

#### Sample size calculation

With an inclusion of 180 patients with MIBC, cT2-T4a N0-N1 M0, at baseline, approximately 150 patients will eventually undergo all procedures of the PRE-PREVENCYS trial including liquid biopsy collection as well as a re-staging TUR during RC. Of these, 30% (45 patients) are expected to have a pCR at RC after NAC. We consider 45 patients a sufficiently large sample for determining the accuracy of individual and/or combined diagnostic tests.

In order to estimate the distribution of 180 patients planned to be included, several assumptions were made:

- After staging with ^18^F-FDG-PET/CT scan, 20 (95% CI 14–26) patients will have disseminated disease and are not candidates for curative NAC and RC.
- After CRE during NAC, approximately 10 (95% CI 6–16) patients will have disseminated disease or progression of local disease after NAC. These patients are either candidates for palliative therapy or immediate RC;
- After CRE, 60 (95% CI 40–90) and 90 (95% CI 70–110) patients will have a complete radiological response or stable disease/partial radiological response, respectively, and are candidates for RC.

#### Data analyses

The CRE will consist of different diagnostic modalities. Descriptive analyses will be performed for all pre-operative clinical, radiological, histopathological variables and on the assessment of mean expression of tissue and liquid biopsy biomarkers. Results of each diagnostic modality will be correlated to the (categorical) pathological ypTN stage in the resection specimen using a Chi-square-based test (categorical-categorical) or a 1-way ANOVA test (continuous-categorical) with post-hoc testing. Multivariate logistic regression analysis will be used to construct a prognostic model for pCR. Suitable candidate biomarkers will be first selected based on established evidence reported in literature. The selected variables will be entered into a ridge logistic regression model, which will be fit using the glmnet R package [27, 28]. Ridge regression is a penalized regression approach that balances model fit (measured by the log-likelihood ratio) and number of parameters (through a penalty on the sum of the squared values of the regression coefficients). Ridge generally leads to a sparse model, where many of the coefficients will be set to values close to zero. Ridge logistic regression requires tuning of one parameter, which will be carried out by 10-fold cross validation using the cv.glmnet() function from the glmnet package. A receiver operating curve (ROC) will be constructed for the final model and the area under the curve (AUC) will be calculated as a measure for discriminative ability. To include all patients in the regression analyses, an imputation procedure of missing values will be performed.

To our opinion, future studies such as a randomized clinical trial comparing NAC followed by RC to NAC followed by close active surveillance in patients with a clinically complete response after NAC are only justified when the predictive model classifies at most 10% of patients with residual ypT1–4 or ypTanyN+ in the resection specimen after RC incorrectly as having a pCR. This will be evaluated in the LASSO logistic regression model with threshold selected by Youden’s J statistic.

#### Ethical and regulatory considerations

The study has been approved by the Medical Ethical Committee of the Amsterdam University Medical Centre (MEC 2019.594) on January 30, 2020 and has been registered in the Netherlands Trial Register (NL8678). The study will be conducted according to the principles of the Declaration of Helsinki (10th version, Fortaleza, 2013) and will be in accordance with the Dutch Medical Research Involving Human Subjects Act (WMO). In each participating centre, the principal investigator will be responsible for recruitment, adherence to the study protocol and follow-up of the included patients. Any other physician of the multidisciplinary team will inform subjects about the study and ask for their consent using information letters and informed consent forms. The project leader (AV) is responsible for the study design, conduct of the trial, preparation of the protocol and revisions and for preparation of case report forms. Revisions of the study protocol will be communicated to all local chief investigators. The project leader will be responsible for data collection and the data master file. Data of each participant will be pseudo anonymized using a subject identification code and put into a comprehensive database using a standardized Web-based Case Record Form (data management system CASTOR EDC) [29]. Patients will not be individually identifiable. An independent data monitoring committee from the Clinical Research Bureau of the Amsterdam UMC will assess the data, according to the Good clinical practice (GCP) requirements. Source data verification will be implemented during the onsite monitoring in observance of original documents. Furthermore, serious complications during re-staging TUR, as described in “Interim analysis and (serious) adverse events”, will be carefully monitored at 5 and 25 inclusions. The final dataset will be available to all study investigators. Given authorships will follow guidelines of the International Committee of Medical Journal Editors [30]. Results will be communicated via international conferences, via publications and via the NTR.

## Discussion

This study is unique and innovative because it aims at predicting a pCR after NAC using a wide set of prognostic variables determined in blood, urine and tissue, on radiological diagnostic examination and on re-staging TUR. Prognostic variables are registered in a large cohort of patients with MIBC at different time points of their treatment.

### Aim

The specific aim of this PRE-PREVENCYS trial is to identify patients who may be potential candidates for a bladder cancer sparing approach and concurrent close active surveillance because they have a high preoperative estimate of pCR**.** The study is considered successful if a predictive algorithm based on clinical, radiological, histopathological variables and expression rates of molecular biomarkers is able to accurately predict the presence of pCR after NAC. The study dictates that no more than 10% of cases in whom pCR was predicted using the prediction algorithm is allowed to have residual ypT1–4 or ypTanyN+ in the RC and ePLND specimen. In this, we propose that residual CIS after NAC and RC is allowed to be missed on CRE. This is, as long as we expect these tumours to be detectable reliably once they have outgrown from non-muscle invasive to MIBC (ypT2–4) during follow-up. During close surveillance, these cases may undergo adjuvant intravesical treatment and (repeated) TUR which may probably result in a long-term disease-control. However, we do propose that residual ypT1–4 or ypTanyN+ should be detected without further delay in order to prevent short-term loss of resectability and to minimize the risk of distant disease dissemination. In case of more than 10% falsely predicted pCR patients, the PREVENCYS trial will be reconsidered.

If this PRE-PREVENCYS trial shows that residual disease after NAC can be determined reliably prior to RC, a randomized controlled trial comparing NAC plus RC versus NAC followed by a close active surveillance protocol will be conducted (the PREVENCYS trial). Hypothetically, this future PREVENCYS trial might result in a bladder sparing treatment for selected patients based on a predictive model, reducing morbidity and mortality, and improving health-related quality of life and reduces health care costs.

### Exclusion

For the present study, only patients with a predominantly muscle-invasive urothelial cell carcinoma of the bladder will be included (cT2–4a). Patients with a large portion of variant histology, such as squamous cell carcinoma, are excluded form study participation because the survival benefit of current cisplatin-based NAC regimens has not been proven in those with other than urothelial cell bladder carcinoma [31]. A further exclusion criterion is the presence of CIS in the prostatic urethra at diagnostic TUR, since the prostatic urethra is a difficult site for re-staging TUR biopsies, especially within a close surveillance protocol. Split-dose cisplatin-based NAC regimens for patients with impaired renal function are allowed within the PRE-PREVENCYS trial as these regimens have been shown non-inferior to conventional regimens in terms of pathological downstaging [20]. Therefore, patients with a CrCl between 40 and 60 ml/min are thus candidates for this split-dose regimen and will be included in this study. Neoadjuvant immunotherapy or immunochemoradiotherapy is not yet considered regular care in patients with MIBC and as a consequence is only given in clinical trials. Patients who undergo any form of immunotherapy are excluded from study participation.

### Pre-operative staging

A ^18^F-FDG-PET/CT is performed for staging purposes prior to NAC, given the incremental value of metabolic information over morphological CT, for assessing lymph node and distant metastases at diagnosis of MIBC [32, 33]. In a recent study, it was reported that a staging ^18^F-FDG-PET/CT after a conventional diagnostic CT thorax and abdomen changed treatment recommendations in 18% of cases, 9% of which changed from potential curative treatment to palliative treatment due to detection of distant metastases [34]. However, the superiority of ^18^F-FDG-PET/CT over conventional CT scanning for clinical response evaluation during NAC has not yet been proven. Currently, ^18^F-FDG-PET/CT is considered not accurate enough to assess response during NAC or to detect pCR after NAC prior to RC [35, 36].

### Outcome of previous non-randomized trials on active surveillance

Several retrospective studies reported on the oncologic outcomes of patients with MIBC who refused or were unfit for subsequent radical surgery. After achieving a clinically complete response to NAC, patients were usually examined by a re-staging TUR. A meta-analysis evaluating a total of 518 patients estimated a 5-year overall survival rate of 72% (95% confidence interval 64–82%) [37]. These survival data are somewhat lower than those reported in patients who experienced a pCR after NAC and subsequent RC, i.e. 80–85% [9]. Apparently, RC adds in the prognosis of patients who undergo NAC by eliminating clinically undetected residual significant disease. This stresses the need for a more accurate model to diagnose for a pCR after NAC. Recently, it was also shown that a re-staging TUR after NAC alone is not adequate enough to predict the response to NAC [38]. In a cohort of 21 patients in whom a standard re-staging TUR was performed after NAC, the sensitivity for the detection of a ypT0 and non-invasive tumour (ypTa) after no visible lesions on cystoscopy was only 61%. Interestingly, this study also retrospectively assessed the expression of two biomarker gene panels of ongoing prospective trials (ClinicalTrials.gov Identifier: NCT02710734 and NCT03609216) in the pre-NAC TUR specimen and reported that the distribution of pathologic staging at RC was not associated with tumour mutation status. However, this study had a retrospective design and the re-staging TUR was not initially applied to assess the feasibility of an active surveillance approach. In addition, not all included patients completed all cisplatin-based NAC cycles.

### Biomarker analysis

Recent studies in small patient cohorts reported mutations in the DDR genes *ATM*, *RB1*, *FANCC*, *ERBB2*, and *ERCC2* to be enriched in patients with MIBC who experienced a pCR after NAC [14–17]. For ERCC2, evidence is accumulating that somatic mutations are a predictive marker of cisplatin-response in urothelial carcinoma [39, 40]. In addition, TMB seems a marker for response to neoadjuvant immunotherapy, which also might hold true for NAC [41, 42]. On the contrary, mismatch repair genes *MSH2* and *MLH1* were recently reported to contribute to cisplatin resistance [43]. Furthermore, urothelial carcinoma can be stratified into luminal and basal tumours based on molecular sub-types [24, 44–46]. Stratification of patients with bladder cancer based on molecular subtype could be an effective strategy for therapeutic regimen allocation. Tumours having a basal subtype are thought to correspond with chemosensitivity in the neoadjuvant setting but a p53-like gene expression signature was shown to be predictive of chemoresistance [47, 48]. An important recent observation was a change in gene expression signature after NAC treatment, which could either be explained by a selection of chemoresistant tumour clones or by NAC induced novel genetic changes causing resistance [49]. This finding underlines the importance of the need for and extensive comparison between pre- and post NAC samples in experimental studies. In addition, a liquid biopsy based test in blood or urine would dismiss the need for repeated invasive procedures and facilitate longitudinal disease and relapse monitoring. For example, EV-microRNAs have been demonstrated to be suitable for cancer diagnosis, treatment response monitoring and early relapse monitoring [50]. Therefore, in this study we will define candidate prognostic and predictive DNA and RNA biomarkers based on results from tissue biomarker studies, and investigate whether a liquid biopsy-based test correlates with or can complement or replace a tissue-based biomarker test.

As of yet, a combination of clinical, radiological and histopathological variables are not yet able to adequately detect the response to NAC in individual patients with MIBC. We hypothesize that by combining these multiple diagnostic modalities in a predictive algorithm that includes the expression of multiple liquid biopsy and tissue biomarkers, a refinement in the selection of patients with MIBC for close active surveillance (bladder-sparing) protocol is feasible.

## Data Availability

Not applicable.

## Abbreviations

CrCl: Creatinine Clearance
CRE: Clinical Response Evaluation
CT: Computed Tomography
CTCAE: Common Terminology Criteria for Adverse Events
DDR: DNA Damage Response
ddMVAC: dose-dense Methotrexate, Vinblastine, Adriamycin and Cisplatin
ePLND: Extended Pelvic Lymph Node Dissection
EV-microRNA: Extracellular Vesicle Next Generation microRNA
^18^F-FDG: 2-Deoxy-2-[18F]fluorodeoxyglucose
Gem/Cis: Gemcitabine/Cisplatin
ISUP: International Society of Urological Pathology
NAC: Neoadjuvant Chemotherapy
NTR: Netherlands Trial Register
NYHA: New York Heart Association Classification
RC: Radical Cystectomy
PET: Positron-Emission Tomography
PIF: Patient Information Form
TEP: Tumour-Educated blood Platelets
TMB: Tumour Mutational Burden
TUR: Transurethral Resection
qPCR: Quantitative Polymerase Chain Reaction
WES: Whole Exome Sequencing
